# Genetic and epigenetic modifications of F1 offspring’s sperm cells following in utero and lactational combined exposure to nicotine and ethanol

**DOI:** 10.1038/s41598-021-91739-6

**Published:** 2021-06-10

**Authors:** Athareh Pabarja, Sepideh Ganjalikhan Hakemi, Elahe Musanejad, Massood Ezzatabadipour, Seyed Noureddin Nematollahi-Mahani, Ali Afgar, Mohammad Reza Afarinesh, Tahereh Haghpanah

**Affiliations:** 1 Neuroscience Research Center, Institute of Neuropharmachology, Kerman University of Medical Sciences, Kerman, Iran; 2 Department of Anatomical Sciences, School of Medicine, Kerman University of Medical Sciences, Kerman, Iran; 3 Research Center for Hydatid Disease in Iran, Kerman University of Medical Sciences, Kerman, Iran

**Keywords:** Developmental biology, Medical research

## Abstract

It is well established that maternal lifestyle during pregnancy and lactation affects the intrauterine programming of F1 offspring. However, despite the co-use of alcohol and nicotine is a common habit, the effects of exposure to both substances on the reproductive system of F1 male offspring and the underlying mechanisms of developmental programming have not been investigated. The present study aimed to examine pre- and postnatal concurrent exposure to these substances on genetic and epigenetic alterations of sperm cells as well as testis properties of F1 offspring compared with exposure to each substance alone. Pregnant dams in the F0 generation randomly received normal saline, nicotine, ethanol, and combinations throughout full gestation and lactation periods. Sperm cells and testes of F1 male offspring were collected at postnatal day 90 for further experiments. High levels of sperm DNA fragmentation were observed in all exposed offspring. Regarding epigenetic alterations, there was a significant increase in the relative transcript abundance of histone deacetylase 1 and 2 in all exposed sperm cells. Moreover, despite a decrease in the expression level of DNA methyltransferase (DNMT) 3A, no marked differences were found in the expression levels of DNMT1 and 3B in any of the exposed sperm cells compared to non-exposed ones. Interestingly, combined exposure had less prominent effects relative to exposure to each substance alone. The changes in the testicular and sperm parameters were compatible with genetic and epigenetic alterations. However, MDA level as an oxidative stress indicator increased in all exposed pups, which may be responsible for such outputs. In conclusion, maternal co-exposure to these substances exhibited epigenotoxicity effects on germline cells of F1 male offspring, although these effects were less marked relative to exposure to each substance alone. These counteracting effects may be explained by cross-tolerance and probably less impairment of the antioxidant defense system.

## Introduction

Despite the well-known hazards of smoking and alcohol drinking during pregnancy, some mothers still continue these practices. Considering the rapid transmission of these substances through the placental membrane^1, 2^ and secretion in breast milk^3, 4^, a significant population still encounter these substances during in utero life and the breastfeeding period.

Each puff of a cigarette is a complex mixture of more than 1000 chemicals, among which, nicotine is well-established as a dangerous substance. Sometimes, nicotine replacement therapy is recommended as an effective method for smoking cessation during pregnancy^5^; however, nicotine exposure by itself affects various body organs, which is a crucial issue. Alcohol intake, especially at higher doses is also associated with symptoms such as growth retardation, facial dysfunction, and brain damage^6^. It is well-established that maternal exposure to nicotine and alcohol could result in abnormalities in the male reproductive system^7–11^ and, subsequently, negatively affect the fertility potential of adult offspring. Nevertheless, there are some contradictory reports^12, 13^.

Epidemiologic studies have shown that the simultaneous consumption of nicotine and alcohol is more common^14, 15^. Approximately eighty percent of people who consume alcohol are smokers^16^. It is well-known that consumption of any of these substances increase the desire for the other^17^. To date, some studies have investigated the effect of prenatal co-exposure to these substances on various tissue, but they have yielded conflicting results^18–21^. However, no study has evaluated the effect of developmental co-exposure to alcohol and nicotine on the testicular properties and sperm parameters of male offspring. Furthermore, it is well-known that maternal exposure to several toxins, accompanied by histopathological changes in the testes, could cause germline genomic damage^22^. High sperm DNA fragmentation could lead to transmission of genetic abnormalities to the embryo and, consequently, affect the health of the next generation^23^. A previous study reported poor sperm DNA integrity in offspring that were prenatally exposed to nicotine alone^24^. However, the effects of prenatally exposure to ethanol alone and combined with nicotine on sperm DNA integrity have not yet been studied.

On the other hand, there is increasing evidence that prenatal exposure to numerous toxins could induce modifications in specific genes expression without altering their sequence via aberrant epigenetic alterations in various tissues of offspring^25–32^. These alterations have permanent adverse developmental and functional effects. DNA methylation and histone modifications are among the major underlying mechanisms of epigenetic changes^33^. These alterations occur through the activity of modification enzymes including DNA methyltransferases (DNMTs), histone acetylates (HAT), and deacetylases (HDACs)^34^. Several studies have evaluated the effects of maternal exposure to nicotine and alcohol alone on a range of epigenetic changes in different tissues, such as somatic and germ cells of male offspring^28, 30, 34^ at the two levels of imprinted genes and epigenetic modification enzymes. For instance, hypomethylation of the placenta^35^, whole fetal genome^36^, and imprinting genes such as H19^37^ as well as decreased DNMT activity^36^ were reported. However, none of the previous studies have evaluated the epigenetic alterations of the sperm cells at the epigenetic modification enzymes level, particularly HDACs, following co-exposure to these substances.

An increasing body of evidence suggests that oxidative stress (OS), induced by an imbalance between ROS production and antioxidant defense, could has a main role in testicular toxicity^38^ and germline aberrant genetic and epigenetic alterations during intrauterine programming^39^. OS status in F1 offspring following nicotine^40^ and ethanol exposure^41^ alone has been reported; however, due to a lack of additive effects of ethanol and nicotine combined on very high levels of ROS production^42^, there is a gap regarding OS status following pre- and postnatal co-exposure to these substances.

Because the co-use of alcohol and nicotine is more common in the world’s population, the present study aimed to investigate the effects of pre- and postnatal co-exposure to both substances on male offspring’s testicular and germline properties, lipid peroxidation, and underling mechanisms of intrauterine programming in F1 male offspring.

## Results

### Biometric analysis: testis mass and dimensions

The values of offspring’s testes parameters are shown in Table 1. Analysis of data revealed that in utero and lactational exposure to nicotine and ethanol, either alone or their combination did not significantly change offspring’s testis mass, length, width and diameter in comparison to the unexposed-vehicle group.

**Table 1:** The effects of exposure to nicotine and ethanol, either alone or simultaneously, during in utero life and breastfeeding periods on testicular weight and dimensions.

Groups	Testis mass (gr)	Testis length (mm)	Testis width (mm)	Testis diameter (mm)
Vehicle	0.12 ± 0.01	7.01 ± 0.13	4.66 ± 0.08	4.56 ± 0.13
Nicotine	0.12 ± 0.01	7.59 ± 0.17	5.06 ± 0.13	4.97 ± 0.13
Ethanol	0.12 ± 0.01	7.12 ± 0.17	4.96 ± 0.06	4.57 ± 0.12
Nic + Ethn	0.11 ± 0.00	7.11 ± 0.14	4.72 ± 0.11	4.61 ± 0.14
Statistics	F(3,18) = 0.21, p = 0.89	F(3,18) = 2.82, p = 0.07	F(3,18) = 3.34, p = 0.05	F(3,18) = 1.95, p = 0.165

### Microscopic and morphometric analysis of seminiferous tubules

#### Microscopic characteristics of seminiferous tubules

**Figure 1:**
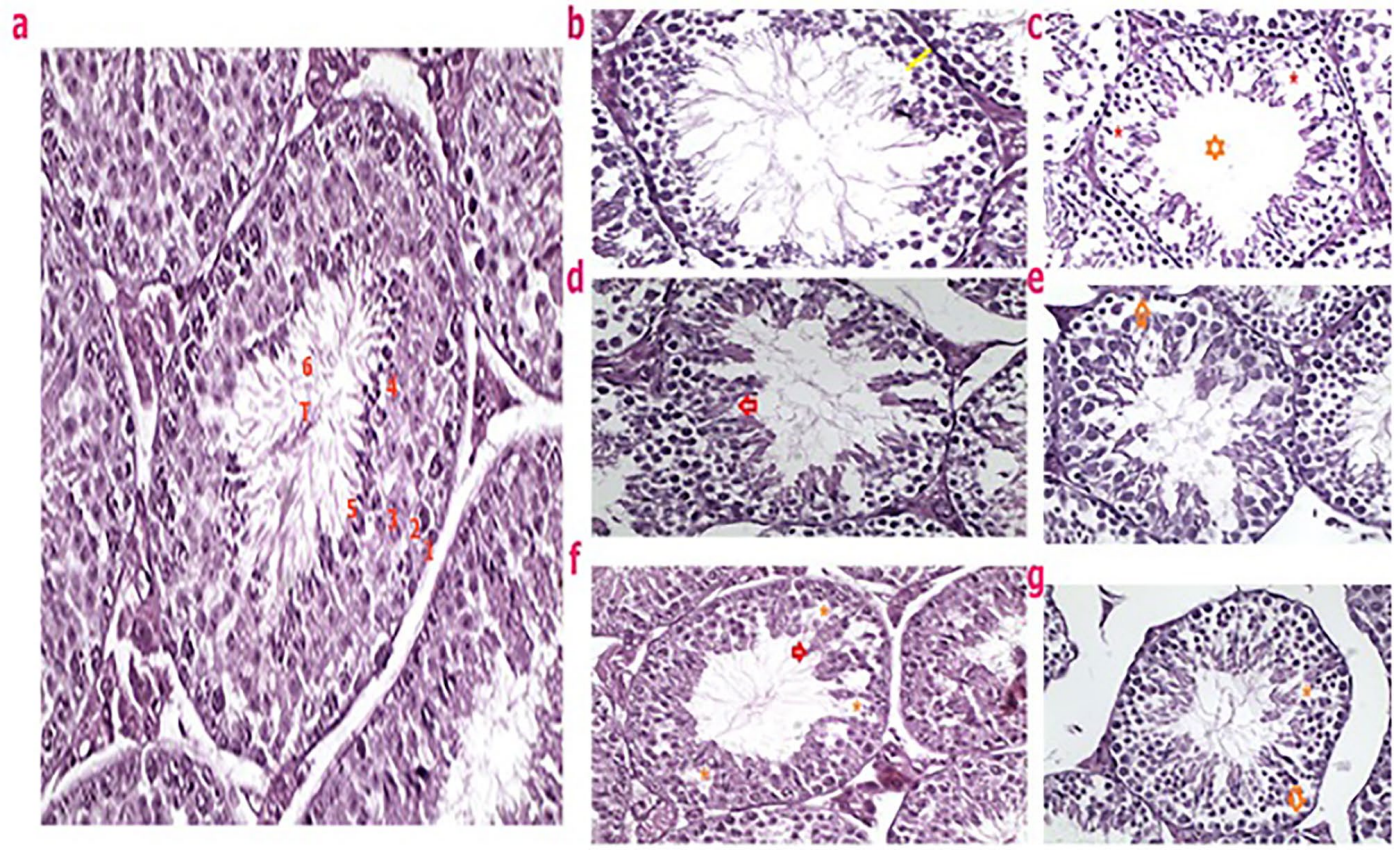
Photomicrographs of testicular sections of adult pups of different experimental groups submitted to the hematoxylin/eosin Y staining. Normal seminiferous tubules (T) with clear lumen and perfect spermatogenesis contained of various germ cells of mice from Vehicle group were observed (**a**). The large quantity of germinal lineage cells in a normal arrangement of cellular types including of spermatogonia (1), primary spermatocyte (2), secondary spermatocyte (3), round spermatid (4), attached sperms (5), released sperm within lumen was observed. Testis of a nicotine-exposed mice (**b**–**e**) showing severe deteriorating change and atrophy of seminiferous tubules (**b**, yellow line), absence (**c**, orange star) or reduction (**d**) of the sperm cells within the lumen of some seminiferous tubules with some degenerative alterations alike vacuoles (**c**, red stars). Note, the absence of germ cells resulting in partial depletion of the seminiferous epithelium and germinal cells atrophy (**d**, red arrows), sloughing germinal epithelium (**d**, red arrow) and detached spermatogenic cells from basement membrane (**e**, orange arrow). Testicular sections obtained from F1 pups of dams exposed to either ethanol (**f**) or concurrent nicotine and ethanol (**g**) showing the restoration of spermatogenic cells in most seminiferous tubules, a few sperm cells in the lumen with several vacuoles in the germinal epithelium (stars).

Histopathological observations of testicular sections of unexposed animals in the vehicle group (Fig. 1a) showed a typical architecture of seminiferous tubules with clear lumens and active spermatogenesis. However, the seminiferous tubules of nicotine-exposed mice (Fig. 1b–e) revealed severe destructions such as atrophy, vacuoles and sloughing germinal epithelium with a small volume of sperm cells in the lumen. Also, in the ethanol (Fig. 1f) and concurrent—exposed animals (Fig. 1g), a perfect spermatogenesis event was observed in almost all the tubules, however several vacuoles were observed and yet sperm cells in the lumen were less than the unexposed mice.

#### Seminiferous tubules diameter

Analysis of seminiferous tubules diameter revealed that pre and postnatal exposure to nicotine and ethanol had a main effect on seminiferous tubules diameter (F_3,18_ = 4.97, p = 0.01). This variable decreased significantly in the nicotine (p = 0.01) and ethanol (p = 0.05) groups in compared to that of the Veh group (Table 2).

**Table 2:** The effects of exposure to nicotine and ethanol, either alone or simultaneously, during in utero life and breastfeeding periods on the averages of Johnsen’s score and seminiferous tubules diameter and area in the testicular tissue of F1 adult pups.

Groups	Seminiferous tubule diameter (μm)	Seminiferous tubule area (µm)^2^	Johnsen’s score
Veh	77.24 ± 0.83	50.985 ± 0.51	9.66 ± 0.04
Nicotine	67.64 ± 1.90^a^**	46.69 ± 2.53	8.98 ± 0.09^a^***
Ethanol	69.59 ± 1.99^a^*	46.29 ± 3.38	9.24 ± 0.08^a^**
Nic + Ethn	72.93 ± 1.96	52.17 ± 1.42	9.23 ± 0.08^a^**
Statistics	F(3,18) = 4.97, p = 0.01	F(3,18) = 1.61, p = 0.23	F(3,18) = 19.38, p = 0.000

Superscript letter (a) indicates a significant difference compared to the vehicle group. *, ** and *** indicate p ≤ 0.05, p ≤ 0.01 and p ≤ 0.001, respectively. Data present Mean ± SEM.

#### Seminiferous tubules area

The results of measuring seminiferous tubules area exhibited that developmental exposure to nicotine and ethanol, either alone or their combination did not change significantly (F_3,18_ = 1.61, p = 0.23) this parameter in the testes of 90 day-old offspring when compared to that of the vehicle group (Table 2).

#### Semi-quantitative analysis of spermatogenesis by Johnsen’s score

The result of Johnsen’s score evaluation showed that exposure to either nicotine, ethanol or their co-exposure during in utero development and lactation periods can change the Johnsen’s score of adult offspring compared to the Veh group (F_3,18_ = 19.38, p = 0.000). This score decreased in the Nic (p = 0.000), Ethn (p = 0.002) and Nic + Ethn (p = 0.002) groups, respectively relative to the Vehicle’s score. However, this parameter was significantly high in the nicotine-exposed pups when compared to the ethanol-exposed pups alone (p = 0.03) and combinations (p = 0.02).

#### Sperm quantitative analysis

The data analysis showed that the administration of either alcohol, nicotine or their co-administration during pregnancy and lactation had main effects on sperm number (F_3, 18_ = 7.57, p = 0.003), motility (F_3, 18_ = 8.54, p = 0.002) and viability (Chi square = 12.58, df = 3, p = 0.006).

#### Sperm number

**Figure 2:**
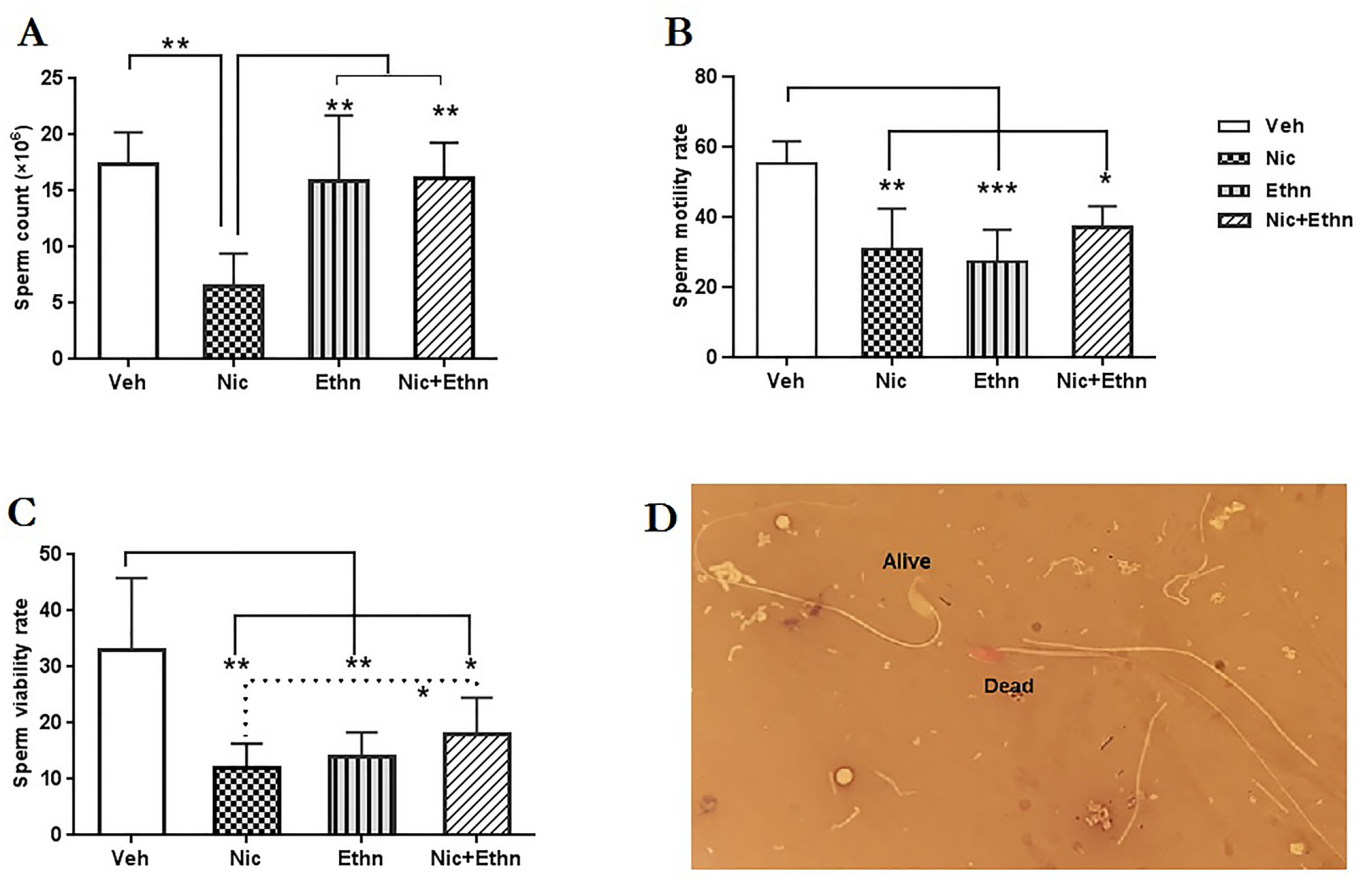
The effect of in utero and lactational exposure to nicotine and ethanol, either alone or concurrently, on sperm parameters of adult offspring. Sperm parameters included of sperm count (**A**), sperm motility (**B**) and sperm vitality (**C**). (**D**) Stained sperm with eosin Y/ nigrosine to determine viability, alive sperm is colorless and dead sperm is pink. *, ** and *** indicate p ≤ 0.05, p ≤ 0.01 and p ≤ 0. 001.

As shown in the Fig. 2A, the sperm number significantly reduced in the Nic group (p = 0.005) when compared to that of the Veh group. However, the sperm number of mice that exposed to nicotine during prenatal and postnatal periods showed a significant difference in compared to those of exposed to either ethanol (p = 0.01) or their combination (p = 0.009) (Fig. 2A).

#### Sperm motility

The mean percentage of sperm motility decreased in all pups that exposed to either nicotine (p = 0.004), ethanol (p = 0.001) alone or concurrently (p = 0.033) when compared to those was observed in the Veh group (Fig. 2B).

#### Sperm viability

Analysis of the sperm viability data showed that there was a significant decrease in the number of viable sperm in the Nic (p = 0.01), Ethn (p = 0.01) and Nic + Ethn (p = 0.03) groups in comparison with the Veh group (31.25 ± 3.69) (Fig. 2C). Also, a marked difference (p = 0.03) in sperm viability rate was observed between the Nic group and Nic + Ethn groups.

#### Sperm DNA fragmentation (SDF)

**Figure 3:**
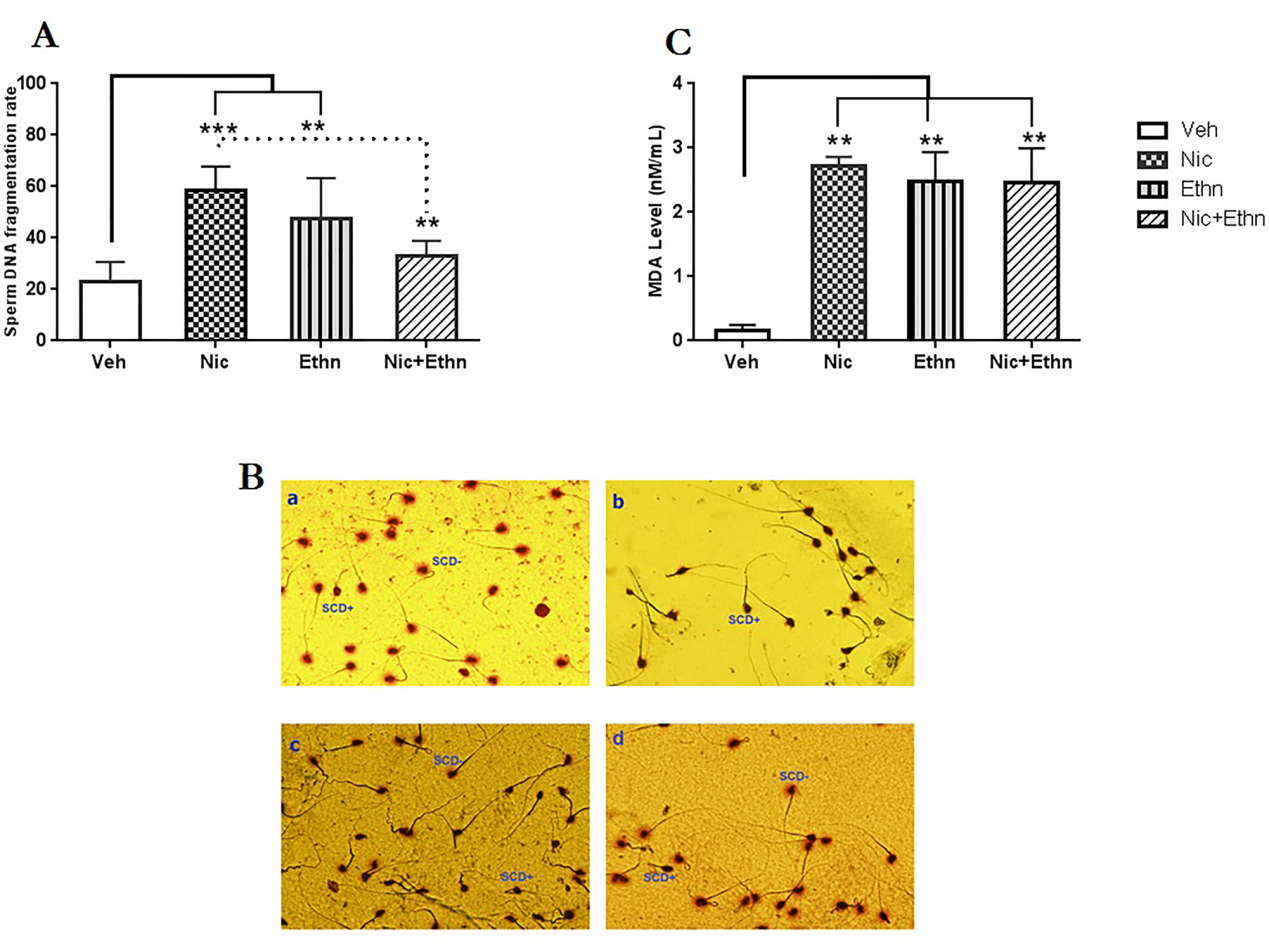
The effects of nicotine and ethanol exposure during embryofoetal development and breastfeeding periods on sperm DNA fragmentation (SDF). (**A**) SDF rate in different experimental groups. (**B**) SDF was assessed by sperm chromatin dispersion (SCD) test. SCD+ indicates sperm cells with fragmented DNA and SCD− indicates sperm cells with normal DNA integrity (×100 eyepiece magnification). Sperm cells of the vehicle (**a**), nicotine-exposed (**b**), ethanol-exposed (**c**) and concurrent exposed (**d**) animals. (**C**) Concentration of malondialdehyde (nmol/mL) in the serum of the mice that exposed to ethanol and nicotine, either alone or concurrently. ** and *** indicate p ≤ 0.01 and p ≤ 0. 001, respectively.

The result of sperm chromatin dispersion test revealed that i*n utero* and lactational exposure had a main effect on SDF level (F_3,18_ = 10.36, p = 0.001). The data is listed in in Fig. 3A,B. A significant increase in the SDF level was observed in the mice that prenatal and lactationally exposed to nicotine (p = 0.001) and alcohol (p = 0.01), alone. Also, a significant difference was found between the Nic and Nic + Ethn groups considering SCD parameter (p = 0.007).

#### Biochemical analysis for oxidative stress biomarker; MDA

A main effect for exposure to either nicotine, ethanol alone or their combination was revealed for malondealdehyde (MDA) level (Chi square = 10.41, df = 3, p = 0.01). This parameter was significantly higher in the serum of all exposed offspring relative to those of the vehicle’ serum (p = 0.01) (Fig. 3C).

### Quantitative RT-PCR analysis for the expression of DNA methylation regulators

**Figure 4:**
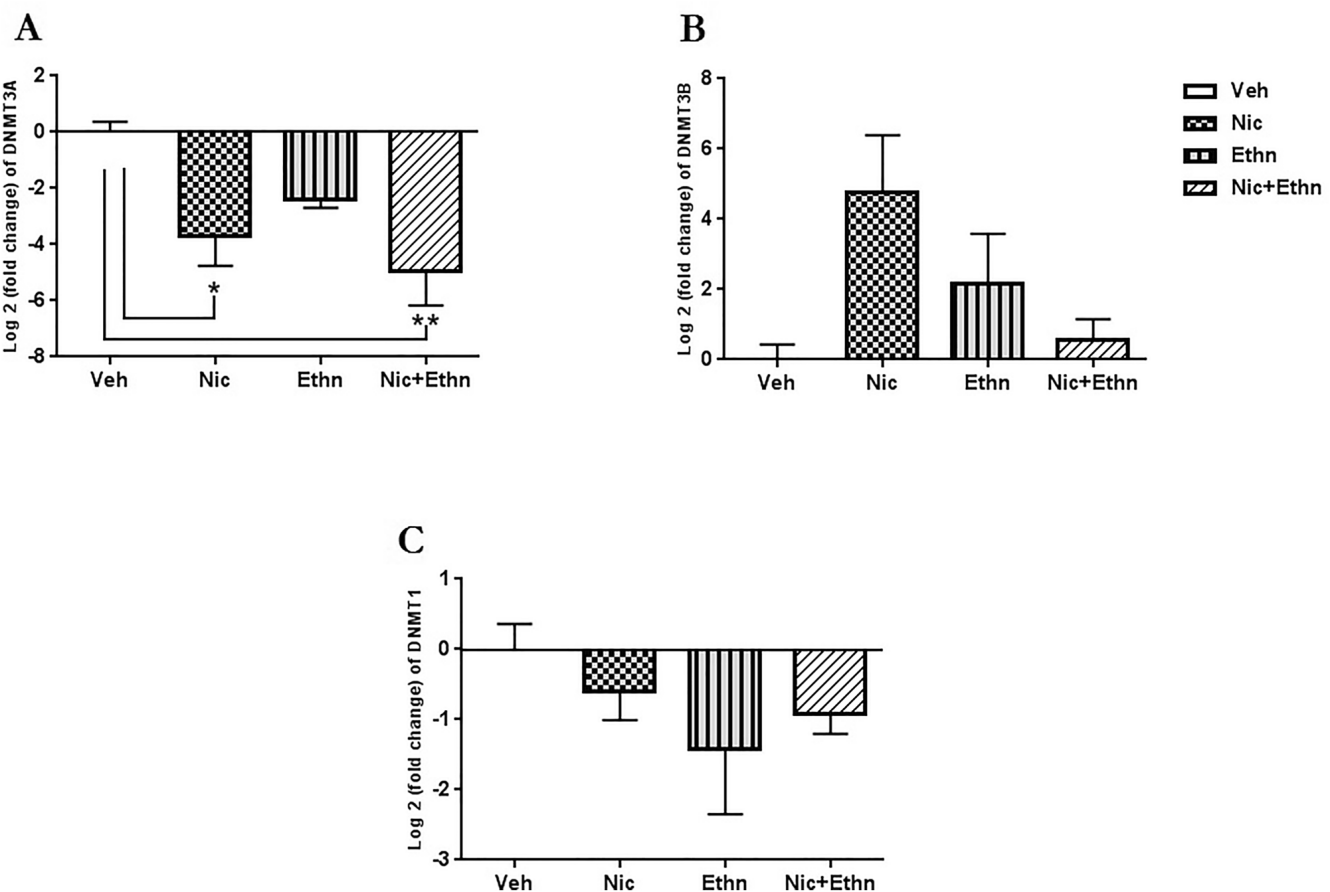
The effect of maternal exposure to nicotine and ethanol, alone and concurrently, on the expression level of enzymes involved in DNA methylation. Real-time quantitative PCR (RT-qPCR) analysis of DNA metyltransferases (DNMT) 1, 3A and 3B gene expression in sperm cells of F1 pups from vehicle ad treated mice. Graphs present mean ± SEM. Data from each gene were normalized to the corresponding value of the housekeeping gene (Actb). Data are exhibited as log twofold changes of relative expression levels of each group compared to the vehicle. * and ** indicate p ≤ 0.05 and p ≤ 0.01.

The expression level of DNMTs (1, 3A and 3B) in the sperm cells of F1 pups was measured and compared to the values of the vehicle group. The data revealed a main effect on the relative abundance of DNM3A mRNA across different groups (F_3, 11_ = 6.41, p = 0.016). In the sperm cells of pups that exposed to nicotine (p = 0.05) alone and also concurrently with ethanol (p = 0.01), DNMT3A expression level was decreased in compared to the vehicle pups. Furthermore, as show in Fig. 4, in utero and lactational exposure to nicotine, ethanol either alone or their combination did not have a main effect on the expression level of DNMT3B (F_3,11_ = 3.404, p = 0.074) and DNMT1 (F_3,11_ = 1.126, p = 0.395).

### Quantitative RT-PCR analysis for the expression of histone hypoacetylation regulators

**Figure 5:**
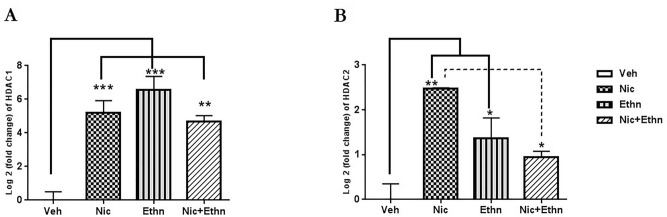
The effect of maternal exposure to nicotine and ethanol, alone and concurrently, on the expression level of enzymes involved in histone deacetylation. Real-time quantitative PCR (RT-qPCR) analysis of histone deacetyltransferase (HDAC) 1 and 2 gene expression in sperm cells of F1 sperm cells from vehicle and exposed animals. Graphs present mean ± SEM. Data from each gene were normalized to the corresponding value of the housekeeping gene (Actb). Data are exhibited as log twofold changes of relative expression levels of each group compared to the vehicle. *, ** and *** indicate p ≤ 0.05, p ≤ 0.01 and p ≤ 0. 001.

As shown in the Fig. 5, prenatal and lactation exposure to nicotine and ethanol, either alone or their combination can change the expression level of HDAC 1 and 2 in the sperm cells of adult offspring compared to the un-exposed mice (F_3, 11_ = 21.28, p = 0.000) and (F_3, 11_ = 12.19, p = 0.003), respectively (Fig. 5). The expression levels of HDAC1 and HDAC2 markedly increased by maternal exposure to nicotine (p = 0.001, p = 0.002) and ethanol (p = 0.000, p = 0.04), either alone or concomitant (p = 0.003, p = 0.178), respectively in comparison with the unexposed mice. However, concurrent-exposed pups showed alleviated changes in the mRNAs expression of histone hypoacetylation regulators compared with the nicotine or alcohol-exposed pups, so that there was a significant difference in the expression level of HDAC2 between the Nic and Nic + Ethn groups (p = 0.02) (Fig. 5).

## Discussion

The development of male gonad and the widespread reprogramming of male gametes in the prenatal development period^43^ makes them sensitive to environmental and chemical factors. To date, many studies have investigated the effects of consumption of nicotine and ethanol alone on the genetic and epigenetic signature of developing fetuses and neonates as well as male gametes. However, the findings are controversial, and the effects of concurrent maternal and lactational exposure to these substances have not yet been elucidated. Therefore, for the first time, this study examined the nuclear DNA integrity and expression levels of a diversity of modifying enzymes, DNMTs and HDACs, in the co-exposed offspring’s sperm cells and compared them with those of offspring exposed to each substance alone. The findings of this study revealed that in utero and lactational exposure to nicotine and ethanol combined led to an increased lipid peroxidation level and altered postnatal sperm and testicular parameters as well as germ cell DNA integrity and epigenome in F1 male offspring. However, exposure to each substance alone had more pronounced destructive effects than those observed in the co-exposed mice. Although the exact mechanism of such ameliorating effects is not yet fully understood, the alteration of peripheral metabolism and pharmacologic effects of one substance with the other may explain these outputs^44^.

The male gamete contains half of the embryo genome. This discloses the high importance of sperm DNA integrity. Several studies have reported that maternal exposure to numerous toxicants may be responsible for DNA damage in mitotic spermatogonia derived from migrated and proliferated primordial germ cells^22^. In agreement with a previous report^7^, high frequencies of sperm DNA damage were observed in all exposed animals in this study, prominently in the nicotine-exposed mice. It is well established that OS is a main cause of genetic material damage in the male germ line^45^. OS occurs when there is an imbalance between reactive oxidizing species (ROS) production and antioxidant defenses. This imbalance was reported in the pregnant mothers^46, 47^ and infants^46–49^ who were exposed to ethanol and smoke. A previous study^50^ reported maternal smoking could reduce antioxidant levels such as vitamin E and A in milk; therefore, the antioxidant status of newborns is also diminished. In the current study, analysis of serum MDA levels as a final lipid peroxidation (LP) product and OS marker showed a significant increase in all exposed offspring. It is assumed that OS resulting from these toxins could affect the genome of germline lineage during early life, leading to disruption of gamete DNA integrity in all exposed adult males.

In addition to assessing the status of sperm genetic material integrity, the current data provides evidence that in utero and lactational exposure to alcohol and nicotine markedly changes male gamete epigenome by significantly increasing HDAC (1 and 2) transcripts and decreasing DNMT3A transcripts in sperm cells. Previous studies have reported that exposure of the fetus to alcohol could change chromatin structure and epigenetic signature in GD 17 developing fetal brain of mice^51^, global DNA hypomethylation of the fetus genome^36^, obvious diminished DNMT activity^35, 36^, hypomethylation of imprinted gene H19, elevated histone 3 acetylation in the lungs^52^ and cardiac cells^53^ as well as reduced HAT activity in the cerebellum^54^. Liu et al.^55^ also showed an increase in HDAC2 expression levels and a decrease in histone 3 lysine 14 (H3K14ac) acetylation in the testicular tissue of F1 pups following prenatal exposure to ethanol (4 mg/kg/daily, 9–20 GD days). A marked increase in the HDACs transcripts in the ethanol-exposed offspring’s sperm cells was also observed in the current study. Gangisetty et al.^56^ demonstrated that in utero exposure to alcohol could lead to pituitary changes via epigenetic mechanisms. In agreement with the current data, they observed a significant increase in HDAC2; however, they also reported a significant increase in pituitary mRNA levels of DNMT 1 and 3b, while no such transcript changes in the sperm cells of exposed pups were observed in the present study. Some studies have explained OS after acute ethanol use^57^ and reduction of methyl donors, such as s-adenosylmethionine (SAM) for DNMTs^58^ as some ethanol mechanisms for the global DNA hypomethylation of sperm cells. In addition, multiple experimental and clinical studies have reported the effects of maternal exposure to smoking on epigenetic alterations in various tissue of exposed fetuses, newborns, children and adolescents. However, no study has yet evaluated the effects of nicotine exposure on the epigenetic modifying enzyme expression levels of sperm cells, especially HDAC1 and 2, that were evaluated in this study. A previous study^59^ determined that second-trimester fetuses of active smoker mothers had different DNA methylation states and a reduction in neuronal proportion in the developing brain prefrontal cortex. They suggested that nicotine is responsible for such effects. Another recent study revealed that in utero exposure to smoking led to differentially DNA methylation in 10,381 CpGs sites of cord blood CD41 positive cells of newborns^25^ as well as 69 CpGs sites^34^ in 36 genomic regions of adult offspring blood cells. Moreover, Rehan et al.^60^ observed that prenatal exposure to nicotine (1 mg/kg) could increase DNA methylation as well as H3 and H4 acetylation in F1 offspring’s testicular cells. They showed a transmission of germline epigenetic marks to F2 offspring. Based on the current data, although differential transcript levels of epigenetic modifying enzymes in the sperm cells of all exposed mice were observed, but it seems that exposure to these substances affects histone deacetylation level more than DNA methylation in the germline. Histone alterations in sperm cells might cause abnormal further generation development, gene expression, and phenotype^61, 62^.

On the other hand, the Johnsen score’s data in the current study showed a decrease in spermatogenesis activity in the testes of alcohol and, particularly, nicotine-exposed pups which are in line with previous studies documenting the transgenerational adverse effects of these substances on spermatogenesis^7, 63, 64^ through several complex mechanisms. As such, deficits in seminiferous tubule organization^7^, disturbed development of Sertoli cells^65, 66^ and their dysmorpho-functions^24^, high prevalence of gonocyte and meiotic germ cell apoptosis^24^ that could lead to fewer numbers of primordial germ cells in the testes of neonates^64^ as well as the spermatogonial stem cell population^24^. However, since spermatogenesis is extremely impressible to abnormal epigenetic alterations^67^ and prenatal exposure-induced established epigenetic changes are suggested as underlying mechanisms of poor spermatogenesis^68^, there seems to be a significant knowledge gap regarding the exact effect of prenatal and lactational exposure to these substances, alone and combination, on the developmental programming of male gonads. As mentioned previously, a recent study^55^ showed that in utero exposure to ethanol resulted in testicular dysplasia in male offspring rats due to increased corticosterone level. The researchers revealed that fetal overexposure to this stress hormone is related to expressive suppression of 3β-hydroxysteroid dehydrogenase (3β-HSD) and testosterone synthesis through epigenetic pathways. Therefore, there is a possibility that high levels of stress hormones following prenatal and lactationally exposure to ethanol and nicotine cause testicular epigenetic changes and subsequently lead to reduced testosterone hormone synthesis. As this sex hormone is necessary for maintenance of normal spermatogenesis quantitatively, its reduction may be one of the mechanisms underlying the disturbed spermatogenesis. However, this hypothesis needs further investigations.

In the present study, consistent with disturbed spermatogenesis, a lower sperm number was observed especially in the nicotine group. In line with the current data, several previous studies have shown a significant reduction in sperm number following prenatal exposure to nicotine and ethanol alone^7, 12^. In the present study a lower sperm motility was also observed in all exposed offspring, particularly the ethanol-exposed pups. However, among sperm parameters, sperm motility^69^ and DNA integrity^70^ are more vulnerable to non-physiologic levels of ROS which were enhanced in all exposed offspring. Sperm membrane is rich in polyunsaturated fatty acids. This characteristic makes it vulnerable to ROS-induced LP. This process can cause structural changes in the sperm membrane and motility apparatus housed within the sperm flagellum as well as alterations in biophysical features such as diminished activity of sodium/potassium ATP, an essential pump for motility^71^. Moreover, since normal mitochondria function is, in part, necessary for sperm motility, and ROS-mediated disturbed mitochondria membrane integrity^72^ could affect its proper function, hence mitochondrial dysfunction in the nicotine-exposed-F1 male^7^ could be a possible reason for decreased sperm motility. Also, a decrease in the expression levels of genes involved in sperm flagellum formation and development or other structures related to motility efficiency might be other possible mechanisms^24^.

However, because OS, a state of misbalance in the lipid peroxidation-antioxidant defense system, can cause significant histopathological changes in the reproductive system^73^ as well as genetic^74^ and epigenetic^39, 75^ modifications, there is increasing evidence that nicotine and ethanol-induced OS may be responsible for these deleterious outcomes in all exposed pups. In addition to lipid peroxidation assessment^40, 76^, many studies have reported antioxidant deficiency in exposed dams and offspring^49, 77^. Although altered antioxidant capacity was not studied in the present research, more destructive effects were observed in the nicotine-exposed mice compared with the ethanol-exposed mice, possibly because of more disturbance in antioxidant defense and, subsequently, more severe OS status. Moreover, nicotine is observed to have a higher level in the fetus bloodstream than that of the smoking mother^78^, and this may be a causal explanation for the more permanent effects of nicotine rather than ethanol. It must be noted that the ethanol effects were applied in a dose-dependent manner^79^; the dosage used in the current experiments was equivalent to that of serum after moderate drinking^80^. Therefore, it seems that ethanol, at this dose, had a slightly more negative effect than nicotine. Also, it is reported that alcohol at low and moderate doses could increase heat shock proteins^81, 82^ which limit inflammation as well as cell death by inhibiting caspase cascade and pro-apoptotic proteins activity^83^.

In the current study, the mild effects of concurrent exposure to these substances on F1 male reproductive properties as well as genetic and epigenetic alterations of male gamete in comparison with the exposure to each one alone were observed for the first time. In line with these findings, a previous study reported an improvement in cognitive impairment in alcohol drinking subjects by smoking^84^. Nicotine has been suggested to be responsible for such alleviating effects^85, 86^. Another study also showed that alcohol toxification is lower in chronic smokers compared to non-smokers^87^. Recently, Bhattacharya et al.^18^ showed that prenatal ethanol exposure (10% v/v) could increase ROS production, LP, and caspases activities and also decrease spatial memory in F1 rats, while simultaneous exposure to ethanol and nicotine (6 mg/kg/day, by mini pump) could alleviate the adverse effects of ethanol on OS and hippocampal memory. However, they exposed pregnant rats to nicotine and ethanol at higher and lower doses, respectively, compared with the current study. Although the exact underlying mechanism for reducing the harmful effects of these substances when consumed together has not yet been elucidated, Hurley et al.^88^ explained that alteration in peripheral metabolism and response of the other^89, 90^ via metabolic and functional cross-tolerance may be involved. Metabolic cross-tolerance happens when extended use of one substance leads to increased metabolism and reduced plasma levels of the other substance, and functional cross-tolerance happens when one substance changes its response to the other drug. Schoedel and Tyndale^44^ reported that ethanol can alter nicotine pharmacological response and nicotine can modify ethanol metabolism. They suggested that alcohol and nicotine co-abuse may increase the elimination of each other. However, assessment of blood concentration of cotinine as a nicotine metabolite and also ethanol in both pregnant dams and their offspring could better clarify the actual exposure levels and confirm this hypothesis. A previous study^42^ reported that oxidative damage and pulmonary histopathological changes were severe in rats that received nicotine alone rather than those animals that received nicotine along with ethanol. Consistent with these results, despite the fact each drug is an individual source of ROS production, the current study observed an enhanced level of lipid peroxidation in the co-exposed mice similar to that observed in the mice exposed to nicotine and ethanol alone. The lack of additive effects of two substances for much higher levels of ROS generation than those observed with each drug alone suggests that these counteracting effects may result in less destructive effects on the antioxidant defense system and consequently lead to fewer alterations in germline cells as well as genetic, epigenetic, and testicular histology compared to themselves individually. Several mechanisms such as modulation of calcium, anti-apoptotic mechanism, and nitric oxide signaling^85^ were also reported to improve the effects between these two determents. Yet, some studies have determined that this counteracting effect was only observed in acute exposure doses^91^. Unlikely, some have reported a synergistic effect^92^. In contrast to the current findings, Sabzalizadeh et al.^19^ revealed that these two substances could synergistically impair cognition in pups. However, it is yet unknown whether such a different effect is dependent on the developing tissue; the interaction of these substances during intrauterine programming might differently affect cells in a developing reproductive system relative to the nervous system. Totally, what was obtained based on this study’s data was that the effects of each substance alone are not completely translatable to the combination state, mostly because of uncertain interactions between diverse chemicals in combination. However, all analyzed parameters in the co-exposed pups were novel, and understanding the underlying mechanisms that co-use of these substances affect fewer than themselves alone represents a very important gap to fill.

In conclusion, for the first time, the present study revealed that in utero and lactational co-exposure to ethanol and nicotine changes the sperm parameters, testicular spermatogenesis, and relative transcript abundance of epigenetic modifying enzymes; DNMT3A, and HDAC 1 and 2 in the sperm cells of F1 offspring. However, these effects were less prominent in the co-exposed mice compared with those observed in the ethanol and nicotine-treated mice alone. The mild effects of combined substances at these particular doses may be explained by cross-tolerance through the decreased metabolism and pharmacologic effects of these two drugs together. Since that nicotine and ethanol-induced OS is one of the main underlying mechanisms of testicular toxicity as well as genetic and epigenetic alterations, therefore the counteractive interactions of these substances and probably, mild changes in antioxidant defense relative to each substance alone may lead to less alterations during the window of developmental programming. More studies are needed to resolve many questions concerning the counteracting effects of these substances. However, based on the genetic and epigenetic changes in male gametes, women should be discouraged from consuming these substances during gestation and lactation.

## Methods

### Animals

A total of 25 adult NMRI mice (19 females and 6 male) aged 8–10 weeks and weighting 25–30 g were used for breeding. These animals were obtained from animal house of Afzalipour faculty of Medicine, Kerman. They were housed in polypropylene cages and were kept under standard condition of animal house with a temperature of 23 + 2 °C and a 12:12-h reverse light/dark cycle; a time-controlled lighting system is used to assurance orderly cycling and the lights coming on at 8:30 a.m. Animals had free access to drinking water and standard pellet food.

### Experiment design

Three female and one male mice (proportion of three females per male) were housed in one cage overnight and the vaginal plug was checked in the following morning. Presence of a vaginal plug was considered as gestational day (GD) 1. The pregnant mice were randomly allocated into four groups and treated daily as follows:

Vehicle group (Veh): the pregnant mice daily received 0.9% normal saline by intraperitoneal (i.p.) injection. Nicotine group (Nic): the pregnant mice daily received nicotine tartrate (1 mg/kg, i.p., free base)^12, 93^. Ethanol group (Ethn): the pregnant mice daily received ethanol (3 g/kg, i.p.)^80^. Nicotine + Ethanol group (Nic + Ethn): the pregnant mice daily received the same doses of nicotine and ethanol, simultaneously^19^.

It has been noted that all drugs were dissolved in 0.9% saline and injected interapertoneally (i.p.) at a volume of 10 mL/kg body weight. Ethanol absolute (MERK, Germany) was also dissolved in 0.9% saline and prepared as a 20% v/v solution eques to 3 g/kg on the base of m_1_v_1_ = m_2_v_2_ formula. All i.p. injections were performed by 26-gauge and 0.5-in. needles, not too deep, in the lower left quadrant of the abdomen at a 30° angle. An aspiration was done to avoid unsuitable location of injection. Treatments started from GD1, continued throughout the pregnancy and lactation periods until offspring weaning (postnatal day; PND 21). Then, the male offspring were maintained in separate cages under the same controlled conditions until PND 90. Only 2 male offspring of each dam were randomly assigned to each experimental group (nine offspring of 5 dams were used for treatment groups; n = 9, and eight offspring of 4 dams were used for vehicle group; n = 8). To exclude the litter effect, we calculated the mean parameters obtained from each sibling per litter for statistical analysis.

### Blood collection

At PND 90, male pups were anesthetized using ketamine (5–10 mg/kg)/xylazine (5 mg/kg) at 8–10 a.m., and their blood were collected from left ventricle. Blood samples were centrifuged at 2500–3000 rpm for 10 min and the serum removed and held at – 20 °C for further evaluations.

### Testis and sperm collection

After blood collection, the anesthetized animal was euthanized by cervical dislocation at the same period of the day (8–10 a.m.), its abdomen wall was cut and the left cauda epididymis of each mice was carefully removed, and transferred into a petri dish containing 1 mL pre-warmed HTF media supplemented by 15 mg/mL bovine serum albumin. It was cut by a pair of scissors, to release sperm cells. The petri dish was incubated at 37 °C for 15 min. Meantime, the left testicle was removed and weighted by a digital scale. Testes dimensions including length, width and thickness, were measured using a standard digital caliper and then fixed by dipping in Bouin’s solution for 48 h.

### Sperm parameters analysis

#### Sperm motility

After incubation of sperm cells for 15 min, the suspension was collected, quietly pipetted, 10 μL of sperm suspension was deposed on the pre-warmed slide, covered by coverslip and then the motility of at least 200 sperm cells was evaluated under a light microscope at 200× magnification in 10 random fields. The motility rate was expressed as the number of motile sperm cells to total counted sperm cells × 100^94^.

#### Sperm number

Five μL of sperm suspension was mixed with 5 μL of sperm fixative solution (formalin/sodium bicarbonate). The mixture was pipetted and the solution was placed on an improved Neubauer hemocytometer and the sperm heads were counted under a light microscope (OLYMPUS BX 51, Tokyo, Japan) at 200× magnification. The sperm number was expressed as million per 1 mL of suspension^95^.

#### Sperm viability

Ten μL of sperm suspension was mixed with an equal volume of 1% eosin Y/5% nigrosin staining solution in a microtube. After 2 min, a smear was prepared, air dried and at least 200 sperm cells were evaluated at 1000× magnification under immersion oil. (Fig. 2-D). The sperm viability rate was calculated as the number of unstained sperm cells (alive) to total counted sperm cells (white + red) × 100^96^. The sperm parameters were evaluated by an expert operator blinded to experimental groups.

#### Sperm DNA fragmentation assessment

Sperm DNA fragmentation (SDF) was assessed by sperm chromatin dispersion (SCD) test based on our previous study with some changes^97, 98^. Briefly, 30 μL sperm suspension was mixed with 70 μL 1% low gelling agarose, placed on a pre-coated slide with standard agarose (0.65%), covered by coverslip and transferred to refrigerator (4 °C) to solidify. After 5 min, the coverslip was carefully removed and the slide was suspended in 0.08 N HCl solution for 17 min at room temperature in darkness. Next, the slide was incubated in two consecutive lysis buffer solutions; lysis buffer solution 1 containing 0.4 M Tris, 0.8 M 2-mercaptoethanol, 1% SDS and 50 mM EDTA (pH 7.5) for 20 min followed by lysis buffer solution 2 containing 0.4 M Tris, 2 M NaCl and 1% SDS, (pH 7.5) for 15 min. The slide was finally incubated in Trisborate–EDTA buffer (0.09 M Trisborate and 0.002 M EDTA (pH 7.5) for 12 min. The slide was washed and dehydrated in increasing concentration of alcohol in water. The slide was stained by Diff-Quick solution for 3 min and air dried. Under light microscopy at 400× magnification, 300 sperm cells were scored based on their halo size; big and medium-sized halo indicate sperm cells without DNA fragmentation, while small-sized halo and also sperm with no halo indicate sperm cells with DNA fragmentation. SDF rate was calculated as the number of sperm cells with small and no halo to total number of counted sperm × 100^99^.

### Testicular morphometric evaluation

The fixed testes were embedded in paraffin and sectioned by a rotary microtome (LEITZ, Germany). Eight 5-μm thick sections of middle part of each testis was prepared at 10-μm intervals. The sections were stained by hematoxylin/eosin, the diameter and the area of 10 round seminiferous tubules in each section were evaluated. In order to evaluate the quality of spermatogenesis and maturity of germinal epithelium, Johnsen’s score was used as a semi-quantitative method in 80 seminiferous tubules per animal^100^. The scores were summed up and their means were reported as Johnsen’s score (JS). All morphometric assessment of testicular tissue (n = 7) was performed in a blind fashion by an expert operator.

### Malondialdehyde (MDA) determination

MDA level as a final product of lipid peroxidation in the serum of exposed pups (seven mice/ group) was assessed by thiobarbituric acid method using Nalondi-Lipid Peroxidation Assay Kit-MDA (NAVANDSALAMAT Company, Urimia, Iran) based on the manufacture instructions. In short, 200 μL of serum was mixed with 1000 μL of working solution containing 2-thiobarbituric acid and trichloroacetic acid and then incubated at 95 °C for 45 min. The mixture was transferred on ice, incubated for 10 min and then, centrifuged at 1500 rpm for 15 min. Absorbance of the supernatant was read at 532 nm by a spectrophotometer (Alpha-1860, Thomas Scientific, USA). MDA concentration was expressed as nM/mL.

### Total RNA extraction and complementary DNA synthesis

Total RNA was extracted from sperm cells by RNX plus solution (CINNAGEN, Iran) based on the manufacturer's instructions. In short, 1 mL of cold RNX plus solution was added to 1 mL of sperm suspension, and incubated at room temperature for 5 min. Then, 200 μL of cold chloroform solution was added, shacked vigorously and centrifuged at 12,000 rpm, at 4 °C, for 15 min. The supernatant was transferred into a clean microtube, equal volume of cold isopropanol solution was added and the microtube was transferred to a − 20 °C freezer, overnight. Next day, the mixture was centrifuged at 12,000 rpm, at 4 °C for 15 min. The upper phase was then removed, the pellet was washed by 75% ethanol, and again centrifuged at 12,000 rpm, at 4 °C, for 15 min. In the next stage, the supernatant was removed and the pellet was placed under a laminar-air flow hood to dryness. The dried pellet was resuspended in 20 μL diethyl pyrocarbonate (DEPC)-treated water. The integrity and quantity of total RNA were verified by 1% agarose gel electrophoresis and the absorbance ratio at 260/280 nm with a NanoDrop 1000 spectrophotometer (THERMO FISHER SCIENTIFIC, Wilmington, DE, United States), respectively. One μg of total RNA was converted into complementary DNA (cDNA) with 1 μL of 200 U/μL SuperScript III Moloney Murine Leukemia Virus (M-MLV) reverse transcriptase (FERMENTASE, Burlington, Canada), Four μL of 5X first-strand buffer, 0.5 μL of 40 U/μL RNase inhibitor, 1 μL oligo (dT) primer and 1 μL 10 mM dNTP were mixed in a 20 μL reaction. The samples were incubated at 42 °C for 60 min, then the enzyme was deactivated at 70 °C for 10 min using a thermocycler (BIOMETRA, Germany)^101^. The specific primers were designed by the primer3 program. The primer sequences are presented in Supplementary table S1. Conventional reverse transcriptase (RT)-PCR was done for optimizing annealing temperature for each primer set. The RT-PCR products were evaluated by agarose gel electrophoresis (2%) (Figure S1).

### Quantitative real time PCR (qRT-PCR)

Due to DNA methylation and histone deacetylation play important roles in the regulation of gene expression of sperm cells, relative mRNA levels of DNA methyltransferases (DNMTs); major enzymes that are responsible for DNA methylation, and also histone deacetylases (HDACs); main enzymes that catalyzed histone deacetylation, were quantified by real-time PCR with SYBR Green Master Mix (GENAXXON BIOSCIENCE, Ulm, Germany) using a Light Cycler Real-Time PCR System (MIC, Queensland, Australia). Beta actin was used as the housekeeping gene to normalize the qRT-PCR reaction. For the amplification reaction, 1 μL synthetized cDNA solution was mixed with a 1 μL specific primer and 10 μL SYBR Green I Master in a 20 μL reaction. The thermocycler cycling conditions was as follows: 95 °C for 15 min (1 rep), followed by 40 cycles of 95 °C for 15 s, 60–62 °C for 60 s and 72 °C for 30 s. Melting curves were checked for the validation of amplification of a single PCR product. The 2^−ΔΔ cycle threshold^ method was used to assess gene expression levels.

### Statistical analysis

Data analysis was conducted by using SPSS, version 16 (https://spss.software.informer.com/16.0/). At first, data was analyzed for normality by using Kolmogorov–Smirnov test. Differences in non-parametric data (sperm viability rate and MDA level) were determined by using Kruskall–Wallis and Mann–Whitney U tests while differences in parametric data (all evaluated variables except those which mentioned above) were determined by One-way analysis of variance (ANOVA) test followed by Tukey post hoc test. The data were presented as mean ± SEM. The GraphPad Prism, version 6 (GraphPad Sofware, San Diego, CA; (https://getpcsoft.wikisend.com/tips/Graphpad_Prism_6_Full_Version_Free_Download.html) as used for graphing. p < 0.05 was considered as a significant difference.

### Study approval

Animal research was approved by the Animal Experiment Committee of the Kerman University of Medical Sciences of Iran (approval no.: EC/97–10/KNRC) and confirming that all animal study methods were conducted in strict accordance with the relevant guidelines and regulations. All experimental procedures were carried out in accordance with the ARRIVE guidelines.

## Supplementary Information

Supplementary Information 1.

## Data Availability

All data generated or analyzed during this study are included in this published article (and its Supplementary Information file).
