# Supplementary material for: Genetic and epigenetic modifications of F1 offspring’s sperm cells following in utero and lactational combined exposure to nicotine and ethanol
Source: Sci Rep. 2021 Jun 10;11:12311. doi: 10.1038/s41598-021-91739-6 (PMC8192516; doi:10.1038/s41598-021-91739-6)
Supplement: Supplementary file 1 — Supplementary Information 1. [file 41598_2021_91739_MOESM1_ESM.pdf]

Supporting information:

Genetic and epigenetic modifications of F1 offspring's sperm cells following *in utero* and lactational combined exposure to nicotine and ethanol

Athareh Pabarja [1], Sepideh Ganjalikhan Hakemi [2], Elahe Musanejad [2], Massood Ezzatabadipour [2], Seyed Noreddin Nematollahi-Mahani [2], Ali Afgar [3], Mohammad Reza Afarinesh [1], Tahereh Haghpanah [2*]

[1] Neuroscience Research Center, Institute of Neuropharmachology, Kerman University of Medical Sciences, Kerman, Iran.

[2] Department of anatomical sciences, School of medicine, Kerman University of Medical Sciences, Kerman, Iran.

[3] Research Center for Hydatid Disease in Iran, Kerman University of Medical Sciences, Kerman, Iran.

Author's email addresses:
Athareh Pabarja: at.pabarja@yahoo.com
Sepideh Ganjalikhan Hakemi: sepideh.ganjalikhan@gmail.com
Elahe Musanejad: e.mn2016@yahoo.com
Massood Ezzatabadipour: ezzatabadipm@gmail.com
Seyed Noreddin Nematollahi-Mahani: nnematollahi@kmu.ac.ir
Ali Afgar: aliafgar1352@gmail.com
Mohammad Reza Afarinesh: Reza.afarinesh@gmail.com
Tahereh Haghpanah: thaghpanah1984@gmail.com

Table S1. The oligonucleotide sequence of primers used for RT-qPCR.

Genes	Gene bank	Gene sequences (5´→3´)	Product size (bp)	Annealing temperature (˚C)
**DNMT1**	NM_001199433.1	F: GGACAGTGACACCCTTTCAGTTG<br>R: CCTTCGTGAAGTGAGCCGTG	95	61
**DNMT3A**	NM_007872.4	F: TCTCAGTGGTGTGTGTGGAGAAG<br>R: GCTTTGCGGTACATGGGCTG	97	61
**DNMT3B**	NM_001271744.1	F: CCAGCCTCACGACAGGAAACA<br>R: CTCCTCATACCCGCTGGCAC	78	62
**HDAC1**	NM_008228.2	F: ACCGTCCTCACAAAGCCAAT<br>R: ACCGTCCTCACAAAGCCAAT	158	60
**HDAC2**	NM_008229.2	F: ACGGGTGGTTCAGTTGCTGG<br>R: AGTCCTCCAGCCCAATTGACAG	77	62.5
**Beta actin (Actb)**	NM_007393.5	F: GTCCACACCCGCCACCAGTT<br>R: GAGCCGTTGTCGACGACCAG	65	63

Figure S1. A representative reverse transcription–polymerase chain reaction (RT-PCR) assay of the expression of epigenetic modifying enzymes in mice sperm cells.

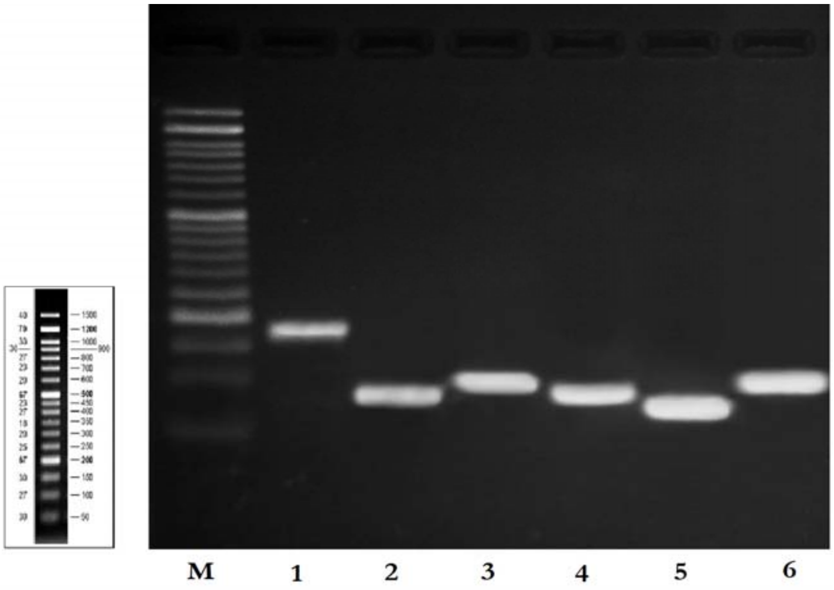

Histone deacetylase 1; HDAC1 (Lane 1) and Histone deacetylase 2; HDAC2 (Lane 2), DNA methyltransferase 1; DNMT1 (Lane 3), DNA methyltransferase 3B; DNMT3B (Lane 4), DNA methyltransferase 3A; DNMT3A (Lane 6). Lane M shows DNA molecular weight markers (50 bp ladder). The housekeeping gene b-actin was used as an internal control (lane 5). Using DNMT 1, DNMT 3A and DNMT3B primers, 95 bp, 97 bp and 78 bp bands corresponding to the expected size for the amplified products of DNMT1, DNMT3A and DNMT3B were detected in mice sperm cells (Lanes 3, 4 and 6). Making use of HDAC1 and HDAC2 primers, 158 bp and 77 bp bands corresponding to the expected size for the PCR products of HDAC1 and HDAC2 were observed in mice sperm cells (Lanes 1 and 2).
